# Epley maneuver versus 360° maneuver for treatment of posterior canal benign paroxysmal positional vertigo in a mechanical rotation chair: a randomized controlled trial

**DOI:** 10.3389/fneur.2026.1909757

**Published:** 2026-09-08

**Authors:** Malene Hentze, Dan Dupont Hougaard, Herman Kingma

**Affiliations:** 1 Balance and Dizziness Center, Department of Otorhinolaryngology, Head and Neck Surgery and Audiology, Aalborg University Hospital, Aalborg, Denmark; 2 Department of Clinical Medicine, Aalborg University, Aalborg, Denmark

**Keywords:** benign paroxysmal positional vertigo, dizziness, Epley’s maneuver, mechanical rotation chair, repositioning chair, TRV-chair, vertigo, vestibular diseases

## Abstract

**Objective:**

To compare the treatment efficacy of the Epley maneuver and the 360° maneuver in a cohort diagnosed with posterior benign paroxysmal positional vertigo (BPPV) using a mechanical rotation chair (MRC).

**Study design:**

Open-label parallel group randomized controlled trial.

**Setting:**

Tertiary university hospital-based outpatient clinic.

**Participants:**

Adults (*n* = 102) diagnosed with unilateral posterior BPPV.

**Intervention(s):**

Participants were randomized to treatment with either the Epley- or the 360° maneuver. Follow-up visits were scheduled biweekly until treatment was successful or up to five treatments were given. By use of questionnaires, patient-reported outcome measures were assessed at baseline and after successful treatment. Subjective recurrences were assessed at 3- and 6-month follow-up questionnaires.

**Main outcome measure(s):**

Primary outcome: treatment success after one treatment. Secondary outcomes: number of treatments to obtain treatment success, Dizziness Handicap Inventory (DHI), visual analogue scale (VAS) of vertigo, maneuver tolerability, and subjective recurrence.

**Results:**

Treatment success after one treatment was comparable between the groups (risk difference (RD): −0.12 (95% confidence interval (CI): −0.32, 0.07)). The cumulative probability of treatment success across treatments showed no clear difference between groups (hazard ratio: 0.77, 95% CI: 0.50, 1.21). Both groups showed clinically meaningful improvements in DHI and VAS of vertigo, with no between-group differences. Subjective recurrence rates were also similar.

**Conclusion:**

Epley- and 360° maneuvers with an MRC demonstrated similar treatment efficacy across objective and patient-reported outcomes, suggesting flexibility in the maneuver choice. Further research should investigate optimization of treatment protocols and long-term follow-up regarding recurrence patterns.

**Clinical trial registration:**

https://clinicaltrials.gov/, identifier NCT05834452.

## 1 Introduction

Benign paroxysmal positional vertigo (BPPV) is the most common cause of peripheral vertigo, with a lifetime incidence of approximately 10% (1). The condition has a peak incidence between 40 and 60 years of age with a female predominance (1–3). Clinically, BPPV is characterized by brief episodes of positional vertigo (often accompanied by nausea) triggered by changes in the head orientation relative to gravity (4–6).

The pathophysiology of BPPV is based on a widely accepted theoretical framework that aligns with clinical observations and biomechanical models. Dislodged otoconia from the utricular macula is believed to migrate into one (or several) of the semicircular canals (SCC), where changes in the head orientation in the plane of the affected SCC(s) will cause them to move, inducing endolymphatic flow and deflection of the cupula, resulting in positional vertigo and an objectifiable positional nystagmus characterized by a typical crescendo-decrescendo pattern and a direction corresponding to the involved SCC(s) (7). Most commonly, the posterior SCC (P-BPPV) is affected (57–72%), followed by the lateral SCC (17–46%), and more rarely the anterior SCC (1–5%) (2, 8, 9). It must be noted that the wide range of prevalence (17–46%) of lateral BPPV might be explained as this diagnosis is easily missed when the manual diagnostic supine roll test (SRT) is performed with insufficient head roll (10). In up to 10% of the cases, multiple SCCs are affected simultaneously (9). Two main subtypes in relation to pathophysiology have been proposed: (1) canalolithiasis, where the otoconia are free-floating within the lumen of the SCC (the most common subtype), and (2) cupulolithiasis, where the otoconia adhere to the cupula, making it heavy (7). While these subtypes might explain the most common clinical findings in relation to BPPV, the exact pathophysiological details and nuances remain incompletely understood.

A diagnosis of BPPV relies on the identification of BPPV-characteristic positional nystagmus during SCC-specific tests, such as the Dix-Hallpike test for P-BPPV (4–6). Treatment of BPPV is done by canalith repositioning maneuvers, which are designed as a sequence of head orientations aiming to guide the dislodged otoconia back into the utricular space. From a biomechanical perspective, the optimal repositioning would require a 360° rotation in the plane of the affected SCC(s). However, such a movement is not feasible when performing manual repositioning maneuvers on a standard examination bed. Therefore, repositioning maneuvers, such as the Epley- (11) and the Semont (12) maneuvers, were developed that move the dislodged otoconia in several steps through the SCC back into the utricular lumen. Predominantly, these maneuvers are performed manually on an examination bed, with reported success rates up to 90% for P-BPPV (13), rising to 100% for the Semont-plus maneuver when combined with self-performed maneuvers at home (14). However, a substantial number of patients require repeated treatments (on average 1.9 and in rare cases more than 10) or might even experience complete treatment failure (15). Moreover, manual repositioning maneuvers may be limited by the patient’s physical cooperation. To mitigate this challenge, mechanical rotation chairs (MRCs) have been introduced, allowing standardized complete 360° rotations in all SCC planes while reducing the dependency on patient cooperation. MRCs are typically combined with videonystagmography goggles to improve the visualization and quantification of the positional nystagmus.

Even though several studies have reported the efficacy of MRCs in both the diagnosis and treatment of BPPV (16–21), current knowledge of their optimal use remains limited. Most frequently, clinical practice and studies use MRC-based treatment maneuvers that are translated directly from the traditional manual maneuvers to the MRC settings. Therefore, the potential of an MRC to perform a near perfect 360° rotation in specific SCC planes, and the effect thereof, have not been fully explored. A recent study by Hougaard et al. compared traditional MRC-based maneuvers (Semont and Epley) with the 360° maneuvers and demonstrated that the Epley maneuver had the highest success rate after a single treatment (18). However, following repeated treatments, no statistically significant difference in the treatment success was detected between the groups (18).

To further address this gap in knowledge (and to reproduce the previous findings), this randomized controlled trial aims to compare the efficacy of the Epley maneuver and the 360° maneuvers for P-BPPV when performed in an MRC. The primary objective was to evaluate treatment success after one treatment. The secondary objectives included the number of treatments required to achieve treatment success, patient-reported outcomes, and recurrence rates.

## 2 Materials and methods

### 2.1 Study design, setting, and participants

This study employed an open-label 2-arm parallel groups randomized controlled trial design, adhering to the Consolidated Standards of Reporting Trials (CONSORT) statement (22) incorporating the principles from the CONSORT extension for nonpharmacologic trials (23). All participants were randomized to receive MRC treatment with either Epley’s maneuver or the 360° maneuver. The randomization was computer-generated and conducted in a 1:1 ratio using permuted blocks of size 4, 6, and 8 (generated with Sealed Envelope Ltd., 2022). The random allocation sequence was implemented (and hidden) in the database (REDCap). Given the nature of the two interventions, blinding of participants or clinicians was not possible. However, data analyses were performed blinded to the allocated treatment maneuver.

The study was conducted between April 12, 2023, and January 9, 2026, at a university hospital-based outpatient clinic specialized in vestibular disorders (the Balance and Dizziness Centre, Department of Otorhinolaryngology, Head and Neck Surgery, Aalborg University Hospital, Denmark).

Participants were referred by general practitioners (North Denmark Region) and private Ear, Nose, and Throat (ENT) clinics (North and Central Denmark Regions). Participants included until January 11, 2024, were offered additional inclusion in a diagnostic randomized trial with crossover as part of their diagnostic examination (the results from this study participation are reported in three separate manuscripts) (10, 17, 24).

Three health care professionals assessed potential participants for eligibility, enrolled them in the study, randomly allocated them to the intervention, performed the interventions, and conducted the follow-up visits. All three clinicians were meticulously trained to diagnose and treat BPPV at the same level as the regular healthcare professionals at the study site (a tertiary, highly specialized center). Furthermore, all study personnel were trained to execute both allocated treatment maneuvers. Supervision was provided by neurotology experts (DDH and HK) throughout the study period. Participants were screened for eligibility at their initial visit. Inclusion criteria included: age of 18 years or above, confirmed unilateral P-BPPV with MRC diagnostics (Bárány Society criteria (4): patient history with positional vertigo and positional BPPV nystagmus during examination), and sufficient Danish proficiency (written and spoken) to understand the informed consent. Exclusion criteria included spontaneous- or gaze-evoked nystagmus, insufficient cooperation with MRC diagnostics and/or -treatment, Inability to attend the scheduled follow-up visits, physical limitations preventing use of an MRC (height above 2 m and/or weight of more than 150 kilograms), recent MRC treatment (within past 6 months), recent intake of sedative antihistamines (within past 7 days), pregnancy, cardiovascular comorbidities (ejection fraction less than 40%, cerebral aneurysm, recent cerebrovascular hemorrhage (within 3 months), and/or arterial dissection disease). There were no changes in eligibility criteria, interventions, outcome measures, or data analysis strategy following initiation of the trial.

### 2.2 Equipment

#### 2.2.1 Mechanical rotation chair

MRC performed with the Thomas Richard-Vitton repositional Chair (TRV-Chair®, Interacoustics©, Middelfart, Denmark). This MRC allows 360° rotations around the pitch-, yaw-, and roll axes by two rotational frames. Lockable preset positions further standardize the participants’ positioning. The participant’s body position in the MRC was secured with a four-point harness, the head was fixed by headrests and straps, and the lower limbs and ankles were fixed by straps. The rotations of the MRC were manually performed by the clinicians.

#### 2.2.2 Videonystagmography

During the MRC treatment, eye monitoring was aided by use of infrared videonystagmography goggles with accompanying software (VF450 and Micromedical VisualEyes version 3.1.0.203, Interacoustics, Middelfart, Denmark) to optimize visualization and recording. To eliminate visual suppression, the goggles were covered during the diagnostic- and treatment maneuvers. The real-time or recorded video images were displayed on a 55-inch wall-mounted screen. Recordings of eye movements allowed a second look by the clinicians and/or supervisors. The software allowed 2D quantification of the horizontal and vertical components of the positional nystagmus [average slow phase velocity (aSPV)].

### 2.3 Intervention

#### 2.3.1 Before the treatment maneuvers

As part of the assessment for eligibility, all participants were screened for spontaneous- and/or gaze-evoked nystagmus and the angular vestibulo-ocular reflex of the lateral SCCs was also evaluated, including a fixation suppression test (manual yaw-axis medium velocity rotation at about 40 degrees/s to the right and left in the MRC with and without visual fixation). Abnormal test results led to exclusion and referral for further evaluation in accordance with local clinical guidelines.

The MRC diagnostic was performed in the following sequence: (1) supine position, (2) right Supine Roll test, (3) left Supine Roll test (the movement between the right and left Supine Roll test was done with one 180° yaw rotation), (4) upright position, (5) right Dix-Hallpike test (6) upright position, and left Dix-Hallpike test. The Supine Roll test was performed before the Dix-Hallpike test to minimize the risk that the Dix-Hallpike test could influence the diagnostic outcome of the subsequent Supine roll test (25). A significant positional BPPV nystagmus for P-BPPV was defined as upbeating vertical nystagmus with a torsional component beating toward the lowermost ear (which was also the affected side) (4), at least five consecutive beats, and an aSPV of a minimum 3°/second (26). The latency and duration of the positional BPPV nystagmus was used to determine whether the BPPV subtype was canalolithiasis (lasting less than 60 s) or cupulolithiasis (lasting more than 60 s). The participants were briefly informed about this study before the diagnostic examination, and detailed study information (written and oral) was provided if a diagnosis of P-BPPV was placed. Participants were offered time to consider participation before providing their written informed consent. All participants gave their written informed consent before the study-specific intervention (treatment in the MRC) was initiated.

#### 2.3.2 Treatment maneuvers

**Figure 1:**
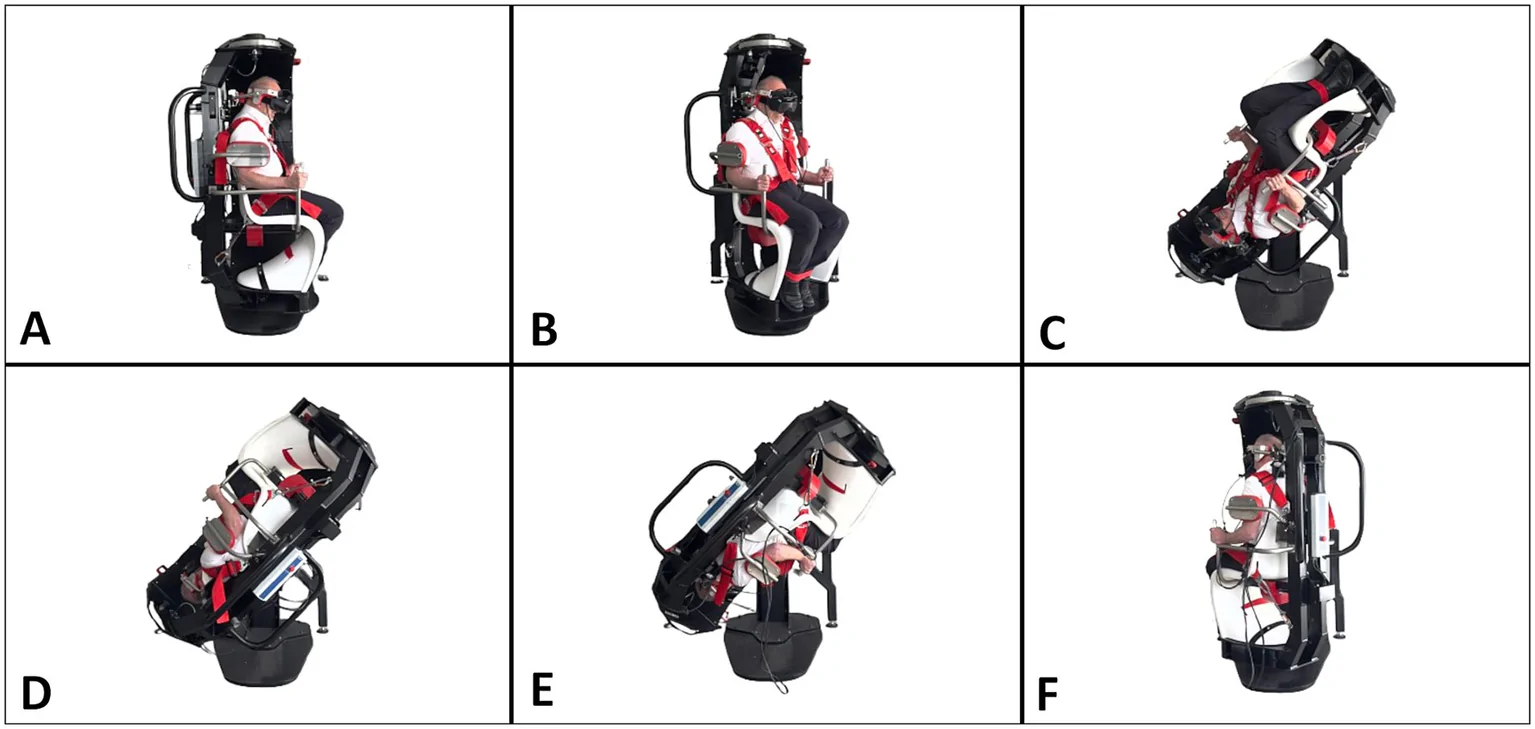
The Epley maneuver in the mechanical rotation chair for right-sided posterior BPPV. **(A)** The participant is seated upright in neutral position. **(B)** The participant is rotated 45° to the right around the yaw axis. **(C)** The participant is rotated 135° backward around the pitch axis. **(D)** The participant is rotated 90° toward the left side around the yaw axis. **(E)** The participant is further rotated 90° toward the left side (a total of 180° yaw rotation). **(F)** The participant is rotated 135° upward around the pitch axis to the upright position. Positions **C–E** were maintained for 30 s from the disappearance of the positional BPPV-nystagmus or for a maximum of 60 s.

**Figure 2:**
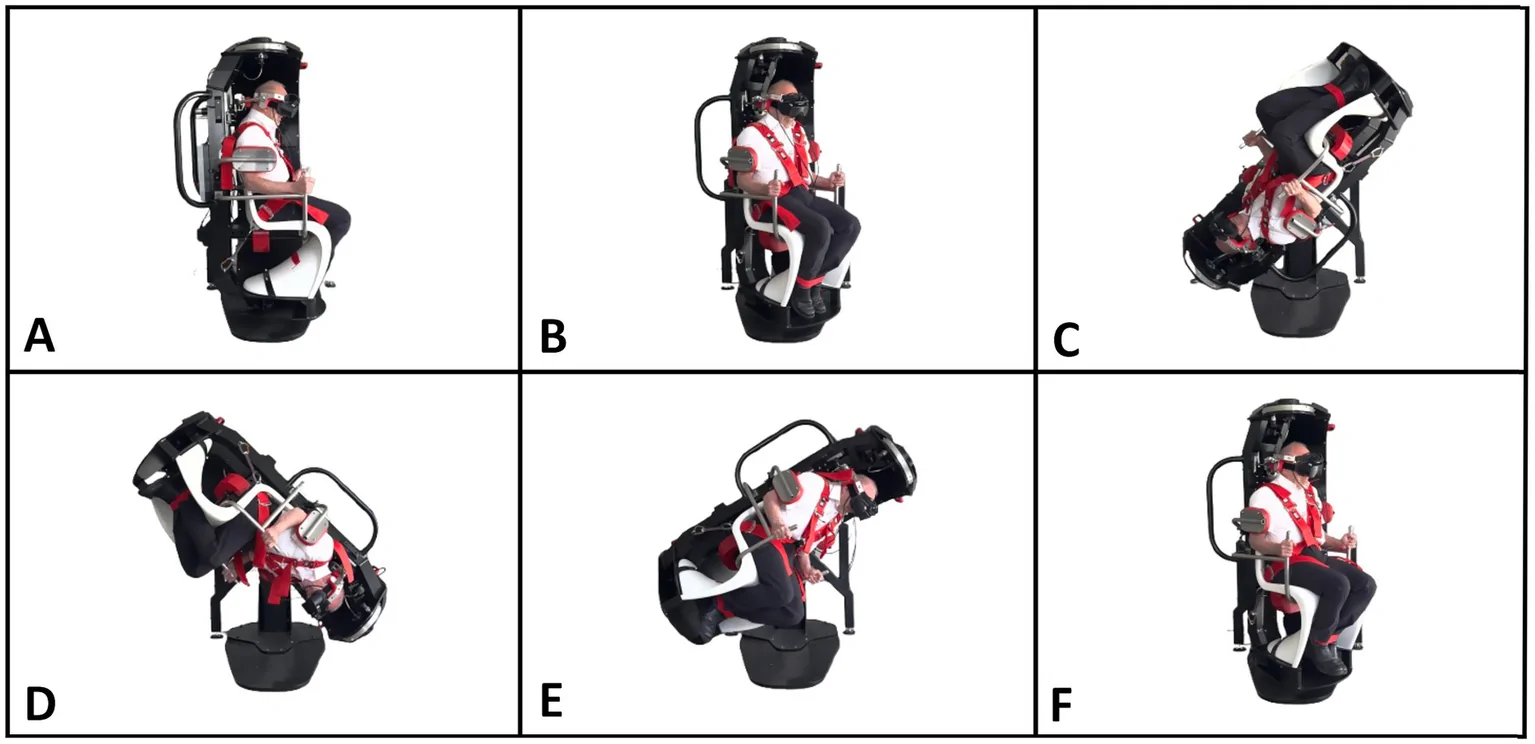
The 360° maneuver in the mechanical rotation chair for right-sided posterior BPPV. **(A)** The participant is seated upright in neutral position. **(B)** The participant is rotated 45° to the right around the yaw axis. **(C)** The participant is rotated 135° backward around the pitch axis. **(D)** The participant is further rotated 90° backward (a total of 225° in the pitch axis). **(E)** The participant is further rotated 90° backward (a total of 315° in the pitch axis). **(F)** The participant is further rotated 45° backward rotation to the upright position (total 360° pitch rotation). Positions **C–E** were maintained for 30 s from the disappearance of the positional BPPV-nystagmus or for a maximum of 60 s.

All individual steps of the Epley- and of the 360° maneuvers are described in detail in Figures 1, 2, respectively. The treatment maneuvers were both designed as three-step procedures to be as similar as possible, except for the individual axes of rotation. The primary treatment positions (Figures 1C–E, 2C–E) with both treatment maneuvers were held for 30 s after the positional nystagmus disappeared or for a maximum of 60 s. The study protocol did not include adding kinetic energy to the maneuvers (i.e., no “bumps” or “impulses”). Adherence to the interventions was ensured by a standardized written description of the procedures at the study site, and no protocol deviations were recorded. Participants received one treatment cycle per treatment session, and no diagnostic examination was performed immediately afterward. No postural restrictions were given following MRC treatments (27) and the participants were carefully instructed to refrain from: (1) performing self-applied liberation maneuvers (Epley- or Semont maneuver) or home exercises (Brandt-Daroff) between treatment sessions, and (2) refrain from the use of sedative antihistamines during the course of MRC treatment(s).

#### 2.3.3 Follow-up visits

Follow-up visits were scheduled every 2 weeks until the baseline P-BPPV was successfully treated or until a maximum of five treatments were given. At all visits, complete BPPV diagnostic examinations, identical to the diagnostics at the time of inclusion, were performed with the MRC. If the baseline unilateral P-BPPV was not successfully treated at the first follow-up visit, then the participant underwent repeated treatment with the same treatment as directed by the initial randomization and was re-scheduled for a new follow-up 2 weeks later. The participants were marked as unsuccessfully treated if: (1) they required more than five treatments and/or (2) a BPPV diagnosis was placed in relation to another SCC than at the initial visit at time of inclusion. With these cases, the participants underwent additional treatment(s) outside the study protocol in accordance with local clinical guidelines.

#### 2.3.4 Participant-reported outcome measures

Before each diagnostic examination (including the initial one at the time of inclusion), participants were asked by the study personnel to rate their vertigo from 0 (no vertigo) to 10 (the worst imaginable vertigo) on a (Visual Analogue Scale (VAS) of vertigo). After the first treatment, participants were asked to rate the comfort and tolerability of the treatment maneuvers on a scale from 0 (comfortable/tolerable) to 10 (uncomfortable/intolerable). The Dizziness Handicap Inventory (DHI) questionnaire was completed at baseline and following successful treatment (28). The Danish version of the DHI-questionnaire, available at the time of initiation of this study, was used (29). The follow-up questionnaire regarding subjective BPPV recurrence (participants were asked whether they had experienced any relapse of BPPV-related symptoms) were completed 3- and 6-month following successful treatment. All questionnaires were distributed by email. For non-responders, a total of three reminders were sent, 1 week apart.

### 2.4 Outcome measures

The primary outcome was the treatment efficacy measured as successful treatment following one treatment. Successful treatment was defined according to the prespecified study protocol as failure to fulfill the Bárány society diagnostic criteria for P-BPPV (4). As these criteria require both positional vertigo in the patient history and positional BPPV nystagmus in the Dix-Hallpike test, resolution of either component was considered sufficient for treatment success. To describe different patterns of treatment response, treatment success was further divided into three subgroups: (1) complete resolution (resolution of both positional vertigo and positional nystagmus), (2) subjective resolution (resolution of positional vertigo despite persisting positional nystagmus), and (3) objective resolution (resolution of positional nystagmus despite persistent positional vertigo). Subjective resolution was defined as a VAS of vertigo score in the patient history of zero or one. Objective resolution was defined as a vertical nystagmus aSPV <3°/second. The secondary outcome measures included: (1) treatment efficacy measured as the total number of treatments required to achieve treatment success, unsuccessful treatment (including refractory BPPV (defined as more than five treatments without successful treatment), canal shift, or new contralateral BPPV), subjective recurrence rate (3 and 6 months after successful treatment), and the participants’ subjective evaluation of the treatment (tolerability, comfort, the subjective treatment effect), and (2) participant-reported outcome measures (difference in baseline and posttreatment scores of DHI and VAS of vertigo).

### 2.5 Data collection

Please refer to Supplementary Table A1 for an overview of data collection points during the study period. Baseline data and data from the follow-up visits were collected from the electronic patient record, patient history, and diagnostic examination. Questionnaires regarding DHI and BPPV recurrences were automatically sent to the individual participant’s email address. All data was managed using REDCap (Research Electronic Data Capture) (version 13.1.37) hosted at a secure server in the North Denmark Region (30, 31).

### 2.6 Sample size estimation

An *a priori* power calculation was conducted with an independent biostatistician. A two-sample, two-sided test of equality was performed with 80% power and a significance level of 0.05. Based on previous studies reporting MRC treatment success rates of 71% for the 360° maneuver (32) and 82% for the Epley maneuver (33), a total of 128 participants (64 in each treatment arm) were required to detect a statistically significant difference between the randomized groups. To account for an anticipated 10% loss to follow-up, the final estimated sample size was therefore 142 participants (71 in each treatment arm).

### 2.7 Statistical analyses

To reflect the study’s focus on treatment efficacy (requiring completion of the intervention), all data analyses were conducted using a per-protocol approach, meaning that participants were included in each analysis only if they met the predefined criteria for that specific outcome measure. Missing data were handled using outcome-specific complete-case analyses without imputation. Therefore, the number of participants included in each analysis varied across outcomes. The specific numbers for analysis were reported in the respective tables. As the proportion of missing data was limited and as the main sources of missing data were non-attendance at follow-up visits or non-responders to questionnaires, no imputation procedures were applied.

Descriptive statistics were used to summarize baseline characteristics. Continuous variables were reported as mean and standard deviation (SD) (or median and interquartile range (IQR) for skewed distributions). Normality was checked visually (histograms and Q-Q plots) and with the Kolmogorov–Smirnov test. Categorical variables were summarized using absolute and relative frequencies. Following the CONSORT guideline, no significance testing was performed for baseline differences between the randomized groups (22).

The primary outcome (treatment success after one treatment) was compared between groups using risk differences with 95% confidence intervals (CIs) and corresponding two-sided *p*-values. Secondary outcomes were analyzed using effect size estimates (with 95% CIs) to describe the difference between the randomized groups. To limit the risk of Type I error due to multiple testing, no hypothesis testing (*p*-values) was done for secondary outcomes. The number of treatments required for treatment success was reported as medians (with interquartile range (IQR)) and compared between the randomized groups using the Cox proportional hazard regression model (Hazard ratios with 95% CI) with treatment success as the event and the treatment number as the time scale. Censoring was done for participants who did not achieve treatment success within five treatments or those who discontinued follow-up (canal shift, new contralateral BPPV, or withdrawal from trial participation). The assumption of proportional hazards was checked with graphical inspection. The cumulative probability of treatment success was visualized using the Kaplan–Meier curve. For binary variables, risk differences (with 95% CI) were reported. For consistency, continuous variables related to participant-reported outcomes were reported as means and SDs, and mean differences (including 95% CIs). For variables with skewed distributions, the means and their difference were obtained by bootstrapping (with 1,000 resamples). To enable the comparison across various scales (i.e., DHI and VAS of vertigo), Cohen’s d was furthermore reported as the standardized mean difference between treatment groups, with the values indicating the effect size [small (*d*: ±0.2), medium (*d*: ±0.5), large (*d*: ±0.8), and very large (*d*: ±1.3)] (34).

The statistical analyses adhered to an *a priori* statistical analysis plan made in collaboration with an independent senior biostatistician and were performed in STATA/MP version 18.0.

## 3 Results

### 3.1 Trial flow, populations for analysis, and baseline characteristics

**Figure 3:**
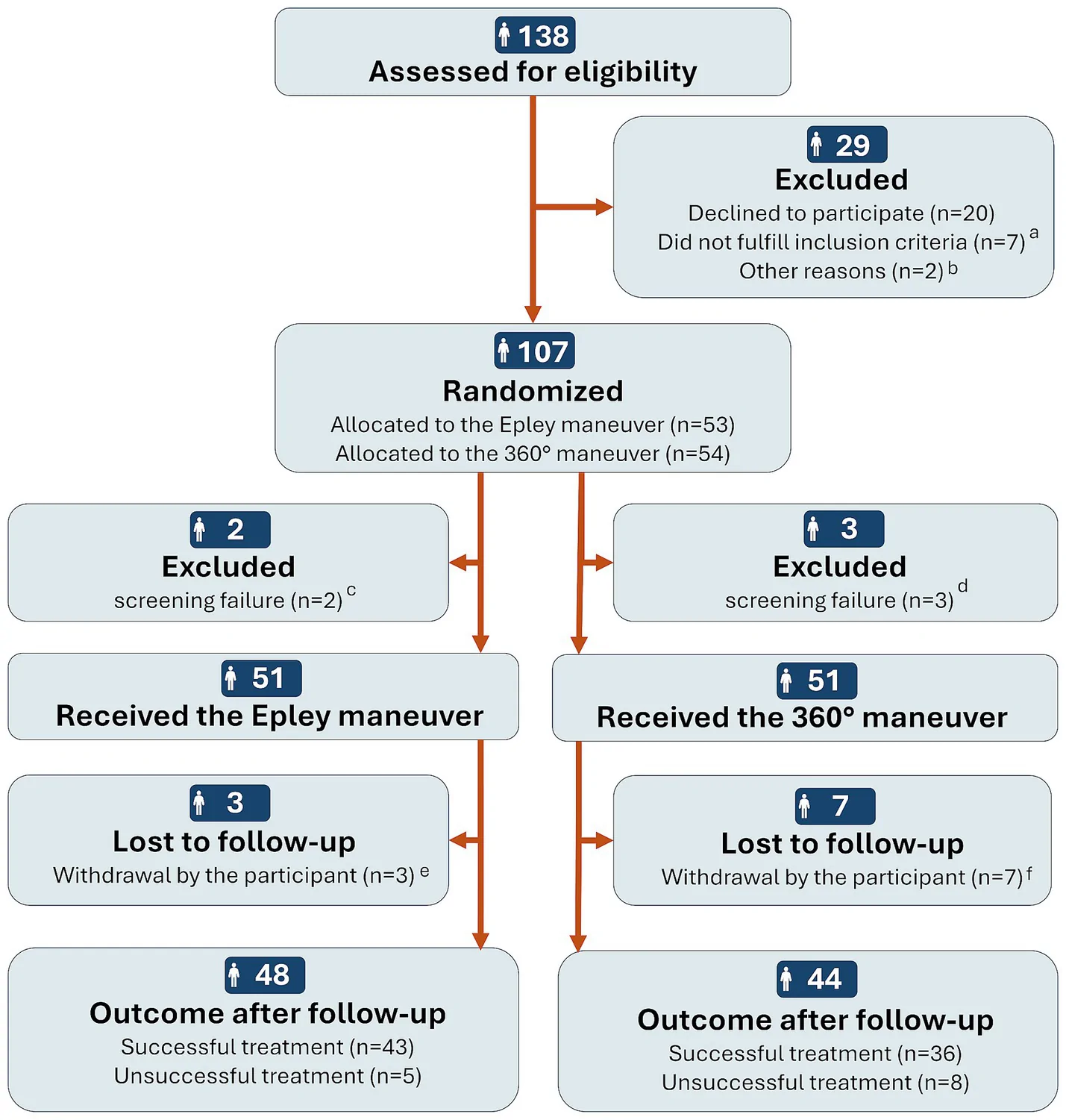
Trial profile. **(a)** Did not fulfill inclusion criteria: bilateral P-BPPV (*n* = 1), ipsilateral multicanal BPPV (*n* = 1), insufficient Danish proficiency (*n* = 1), not possible to attend follow-up (*n* = 1), recent cerebrovascular event (*n* = 1), spontaneous nystagmus (*n* = 1), insufficient cooperation in the MRC (*n* = 1). **(b)** Other reasons for exclusion: complete voice rest (*n* = 1), wrongly excluded (*n* = 1). **(c)** Screening failure: ipsilateral multicanal BPPV (*n* = 1), bilateral BPPV (*n* = 1). **(d)** Screening failure: participant duplication (*n* = 1), recent intake of antihistamines (*n* = 1), bilateral P-BPPV (*n* = 1). **(e)** Lost to follow-up: not possible to attend follow-up (*n* = 1), remission of vertigo (*n* = 1), unknown reason (*n* = 1). **(f)** Lost to follow-up: remission of vertigo (*n* = 4), unknown reason (*n* = 2), participant wanted another treatment in the MRC (*n* = 1), unknown reason (*n* = 1). Analyses were performed per protocol. The number of participants included in each outcome analysis varied according to missing data (please refer to Tables 2, 3, 4). Reasons for unsuccessful treatment are detailed in Table 3.

Please refer to Figure 3 for a detailed description of the trial profile. A total of 138 participants with P-BPPV were assessed for eligibility, and 107 (78%) with unilateral P-BPPV were included and randomized to treatment with either the Epley maneuver or the 360° maneuver, both performed with the MRC. The main reason (*n* = 20/29, 69%) for exclusion was declining to participate (mainly due to logistical reasons in relation to the follow-up scheme of the study). Other reasons for exclusions are elaborated in the legend of Figure 3. Due to screening failure (explicitly reported in the legend of Figure 3), an additional five participants (randomized to Epley maneuver: 2, randomized to 360° maneuver: 3) did not receive the allocated intervention. Following this, baseline data was available for 51 participants in the Epley group and 51 in the 360° group (Table 1). For the primary outcome measure (treatment success following one treatment), 48 participants in the Epley group and 46 participants in the 360° group showed up for the first follow-up visit and were included in the analysis. For the secondary outcomes, analyses were performed in the outcome-specific complete-case populations. The number of participants is reported in Figure 3 and Tables 2, 3. Reasons for exclusions and losses to follow-up are described in Figure 3.

**Table 1:** Baseline demographic and characteristics of participants with posterior canal BPPV.

	Total (*n* = 102)		Epley maneuver (*n* = 51)		360° maneuver (*n* = 51)	
Age (year), Mean ± SD	57.1	±14.3	54.8	±12.75	59.59	±15.35
Female sex, *n* (%)	71	(69.6)	40	(78.4)	31	(60.6)
Referred by						
General practitioner, *n* (%)	78	(76.5)	39	(76.5)	39	(76.5)
Private ENT clinic, *n* (%)	24	(23.5)	12	(23.5)	12	(23.5)
Symptom duration (days), median [IQR]	39.5	[18, 93]	42	[21, 108]	35	[14, 91]
Posterior BPPV characteristics						
Affected side						
Right side, *n* (%)	58	(56.9)	31	(60.8)	27	(52.9)
Left side, *n* (%)	44	(43.1)	20	(39.2)	24	(47.1)
Posterior BPPV subtype						
Canalolithiasis, *n* (%)	94	(92.2)	47	(92.2)	47	(92.2)
Cupulolithiasis, *n* (%)	8	(7.8)	4	(7.8)	4	(7.8)
Idiopathic BPPV, *n* (%)	87	(85.3)	42	(92.4)	45	(88.2)
Secondary BPPV*, *n* (%)	15	(14.7)	9	(17.7)	6	(11.8)

SD, standard deviation; IQR, interquartile range; ENT, Ear-Nose-Throat. *Secondary BPPV includes recent head trauma (<6 months), ipsilateral vestibular neuritis, ipsilateral sudden deafness, Meniere’s disease and/or ipsilateral inner ear surgery. The table includes data from participants (*n* = 102) who received the allocated treatment maneuver at baseline. Percentages may not sum up to 100 due to rounding off. Please note that baseline characteristics were comparable between the two randomized subgroups, with no clinically relevant imbalances observed.

**Table 2:** Treatment success following one treatment maneuver.

	Epley maneuver (*n* = 48)		360° maneuver (*n* = 46)		Risk difference	(95% CI)	*p*-value
Treatment success after one treatment							
Success*, *n* (%)	32	(66.7)	25	(54.4)	−0.12	(−0.32, 0.07)	0.22
No success, *n* (%)	16	(33.3)	21	(45.7)	–		
Among participants with treatment success							
Complete resolution^a^, *n* (%)	25/32	(78.1)	20/25	(80.0)	−0.09	(−0.29, 0.12)	0.40
Subjective resolution only^a^, *n* (%)	2/32	(6.3)	1/25	(4.0)	−0.02	(−0.09, 0.05)	0.58
Objective resolution only^a^, *n* (%)	5/32	(15.6)	4/25	(16.0)	−0.02	(−0.14, 0.10)	0.78

CI, confidence interval. *Treatment success was defined as when BPPV was not diagnosed according the Bárány Society criteria (4). ^a^Subjective resolution was defined as a Visual Analogue Scale of vertigo of 0–10, and an objective resolution was defined as an average slow phase velocity of <3°/second of the baseline average slow phase velocity of vertical nystagmus. Complete resolution included both subjective and objective resolution. All risk differences were calculated with the Epley maneuver as reference. Percentages may not sum up to 100 due to rounding off. Please note that no statistically significant differences were observed for treatment success after one treatment between the two randomized subgroups.

**Table 3:** Treatment course and recurrence.

	Epley maneuver (*n* = 48)		360° maneuver (*n* = 46)		Effect size for the intergroup difference	(95% CI)
Number of treatments until treatment success						
Mean ± SD*	1.44	±0.92	1.58	±1.06	–	–
Median [IQR]	1	[1, 1]	1	[1, 2]	0.77^a^	(0.50, 1.21)
Accumulated treatment success						
1. Treatment, *n* (%)	32	(66.7)	25	(54.4)	−0.12^b^	(−0.32, 0.07)
2. Treatments (cumulative), *n* (%)	39	(81.3)	31	(67.4)	−0.14^b^	(−0.31, 0.04)
3. Treatments (cumulative), *n* (%)	41	(85.4)	34	(73.9)	−0.12^b^	(−0.28, 0.05)
4. Treatments (cumulative), *n* (%)	42	(87.5)	35	(76.1)	−0.11^b^	(−0.27, 0.04)
5. Treatments (cumulative), *n* (%)	43	(89.6)	36	(78.3)	−0.11^b^	(−0.26, 0.03)
Unsuccessful treatment						
Refractory BPPV^†^, *n* (%)	1	(2.1)	2	(4.4)	0.02^b^	(−0.05, 0.09)
Canal shift, *n* (%)	1	(2.1)	3	(6.5)	0.04^b^	(−0.04, 0.13)
New contralateral BPPV, *n* (%)	3	(6.3)	3	(6.5)	0.00^b^	(−0.10, 0.10)
Subjective BPPV recurrence	(*n* = 38)		(*n* = 30)			
3 months, *n* (%)	18	(47.4)	10	(33.3)	−0.14^b^	(−0.37, 0.09)
6 months (cumulative), *n* (%)	22	(57.9)	14	(46.7)	−0.11^b^	(−0.35, 0.13)
	(*n* = 22)		(*n* = 13)			
Time to recurrence (days), Mean ± SD	72.86	±50.69	69.15	±51.47	3.72^c^	(−32.57, 40.00)

SD, standard deviation; IQR, interquartile range. *Continuous variables with skewed distribution: mean ± SD were obtained using bootstrap (with 1,000 resamples). ^†^No treatment success registered within five treatments. The recurrence outcome was assessed only among participants who achieved treatment success and had available follow-up data. Effect size estimates for intergroup differences were reported as Hazard ratio (95% CI)^a^ for the number of treatments to successful treatment, risk difference (95% CI)^b^ for binary variables, and mean difference (95% CI)^c^ for continuous variables with normal distribution. All effect sizes were reported with the Epley maneuver as the reference. Please note the pattern suggesting fewer treatments to achieve treatment success in the Epley group and a higher proportion of participants reporting subjective recurrence in the Epley group compared to the 360° group. These observations should be interpreted with caution, as that all effect size estimates had CIs that included zero, indicating no clear difference between the treatment groups.

Baseline characteristics were comparable between the randomized groups (Table 1). The study population predominantly consisted of females (*n* = 71/102, 70%), with a mean age of 57.1 ± 14.3 years (range: 26–88 years). Most participants were referred by their general practitioner (*n* = 78/102, 76%), while 24/102 (24%) were referred by a private ENT clinic. Most cases (*n* = 87/102, 85%) were diagnosed with idiopathic unilateral P-BPPV, with canalolithiasis being the most frequent BPPV subtype (*n* = 94/102, 92%).

### 3.2 Primary outcome: treatment success after one treatment

Treatment success after one treatment occurred in 32 out of 48 (67%) of participants in the Epley group and 25 out of 46 (54%) in the 360° group, corresponding to a risk difference of −0.12 (95% CI: −0.32, 0.07, *p* = 0.22) (Table 2). Among participants with treatment success after one treatment, the majority (Epley: *n* = 25/32, 78%; 360°: *n* = 20/25, 80%) had complete resolution (both subjective and objective). A smaller proportion of participants had subjective-only (Epley: *n* = 2/32, 6%; 360°: *n* = 1/25, 4%) or objective-only resolution (Epley: *n* = 5/32, 16%; 360°: *n* = 4/25, 16%) of the P-BPPV (Table 2). Among those with subjective resolution only (*n* = 3), the recorded aSPV was 4–6, and among those with objective resolution only (*n* = 9), the reported VAS of vertigo was 2–3 for the majority of cases (*n* = 7).

### 3.3 Secondary outcomes

#### 3.3.1 Number of treatments to treatment success

**Figure 4:**
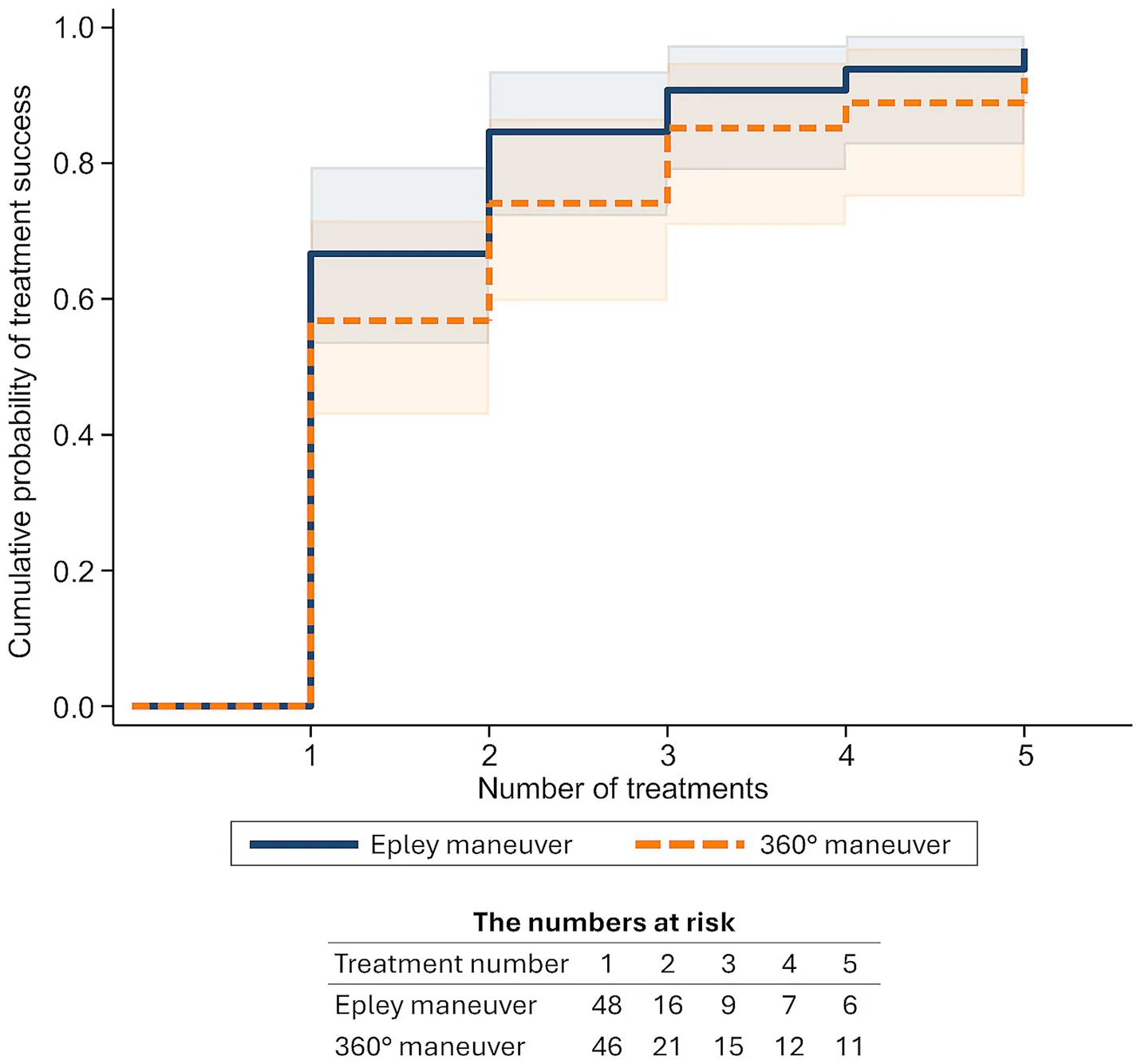
Kaplan–Meier curve of cumulative treatment success by number of treatments. The cumulative probability of treatment success (y-axis) is shown as a function of the number of treatments received (x-axis) for participants randomized to the Epley maneuver (solid blue line) and the 360° maneuver (dashed orange line). The shaded areas illustrate the 95% confidence intervals. Participants were censored if treatment success was not achieved within five treatments or if follow-up was discontinued due to canal shift or new contralateral BPPV during the treatment course. The numbers below the x-axis indicate the number of participants at risk before each treatment. Please note that the 95% confidence intervals overlap for every treatment number, suggesting no difference in the probability of treatment success between groups. A minor separation of the curves is observed after one treatment, with more participants remaining at risk in the 360° group. However, this observation should be interpreted with caution due to small numbers. Please refer to Table 3 for the corresponding hazard ratio (including its 95% confidence interval).

During the treatment course, most participants were successfully treated with the allocated treatment maneuver (Epley: *n* = 43/48, 90%; 360°: *n* = 36/46, 78%). Risk difference: −0.11 (95% CI: −0.26, 0.03). The number of treatments required before treatment success is summarized in Table 3. The median number of treatments was 1 [IQR: 1, 1] for the Epley group and 1 [IQR: 1, 2] for the 360° group (corresponding to bootstrapped means of 1.44 ± 0.92 for the Epley group and 1.58 ± 1.06 for the 360° group). Cox regression analysis revealed a Hazard ratio of 0.77 (95% CI: 0.50, 1.21) for treatment success of the 360° maneuver when compared to the Epley maneuver. Kaplan–Meier curves demonstrated similar cumulative probabilities of treatment success across treatment sessions in both groups (Figure 4).

#### 3.3.2 Unsuccessful treatments: refractory BPPV or a change in BPPV localization

Refractory BPPV occurred in one out of 48 (2%) in the Epley group and in 2/46 (4%) in the 360° group (Table 3). In each sub group, a small number of participants experienced canal shift (Epley: *n* = 1/48, 2%; 360°: *n* = 2/3, 7%) and new contralateral BPPV (Epley: *n* = 3/48, 6%; 360°: *n* = 3/46, 7%) were observed during the treatment course (Table 3).

#### 3.3.3 Subjective recurrence

Among the participants with successful treatment and available follow-up data, subjective recurrence (positional vertigo) was reported by 22 out of 38 (58%) in the Epley group and by 14 out of 30 (47%) in the 360°, with a corresponding risk difference of −0.11 (95% CI: −0.35, 0.13) (Table 3). The mean time from the time of successful treatment to subjective recurrence among participants with reported subjective recurrence was 72.9 ± 50.7 days for the Epley group and 69.2 ± 51.5 for the 360° group. Of participants with reported subjective recurrence(s), only 10 out of 36 (28%) reported that they received treatment from a healthcare professional.

#### 3.3.4 Participant-reported outcomes

**Figure 5:**
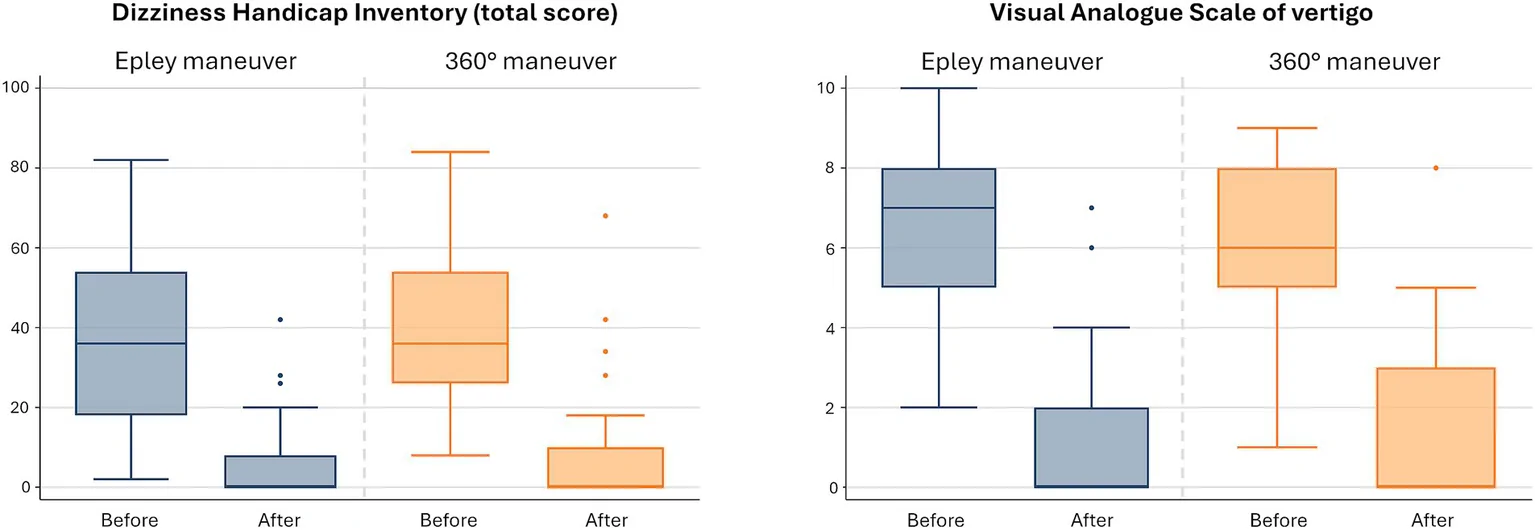
Individual changes in participant-reported outcome measures. The figure shows individual changes in the total score of Dizziness Handicap Inventory (DHI) and Visual Analogue Scale (VAS) of vertigo before (baseline) and after successful treatment. Each line represents an individual participant, connecting the paired baseline and post-treatment scores. Data are presented separately for the Epley (blue) and the 360° maneuver (orange). Please note that most participants’ posttreatment scores reached zero in both treatment groups and for both outcome measures. DHI, Dizziness Handicap Inventory; VAS, Visual Analogue Scale.

**Figure 6:**
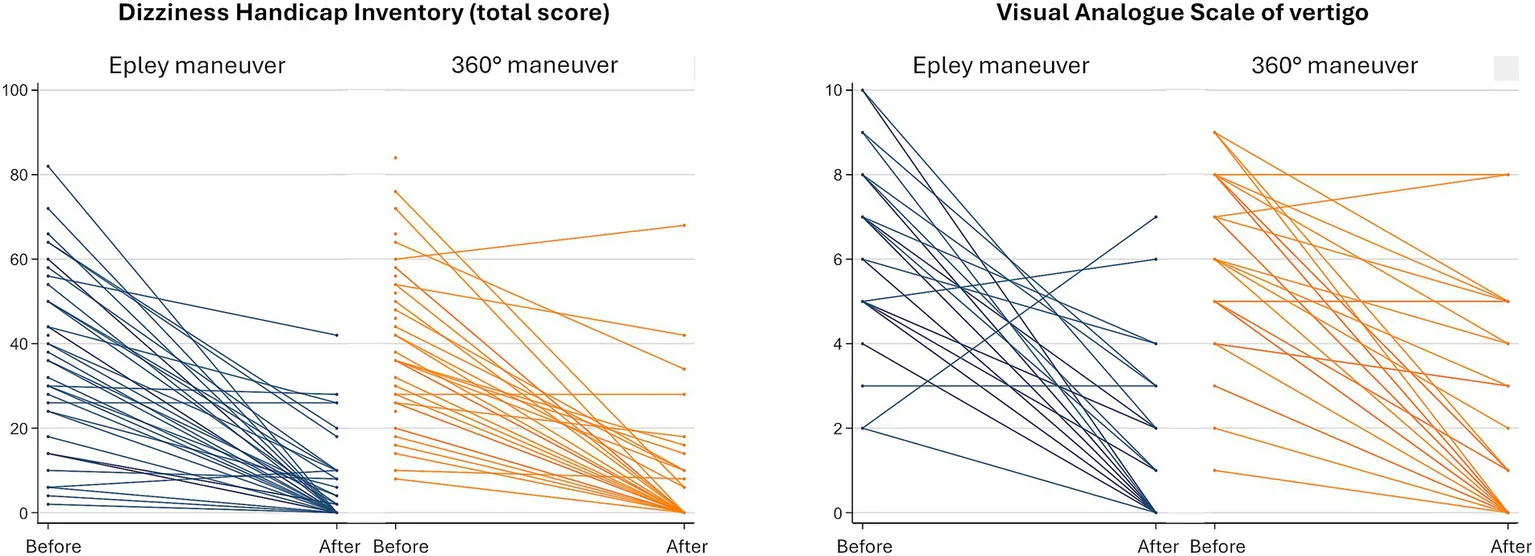
Distribution of participant-reported outcome measures. The figure shows the distribution of the total score of Dizziness Handicap Inventory (DHI) and Visual Analogue Scale (VAS) of vertigo before (baseline) and after successful treatment. The boxplots are presented separately for the Epley maneuver (blue) and the 360° maneuver (orange). Both measures (DHI and VAS of vertigo) have boxplots of the score at before (baseline) and after successful treatment. In each boxplot, the center line represents the median, the upper and lower limits of the box represent the interquartile range (IQR), the whiskers represent values within 1.5 times the IQR, and the dots represent outliers. Please note that post-treatment scores are clustered with a median of zero in both treatment groups and for both outcome measures. DHI, Dizziness Handicap Inventory; VAS, Visual Analogue Scale; IQR, Interquartile range.

Participant-reported outcome measures are presented in Table 4 and Figures 5, 6. At baseline, the total DHI-score and VAS of vertigo were similar across the treatment groups. Following successful treatment, both groups reported improvements in the total DHI-score and the VAS of vertigo score. The mean change in total DHI-scores was −29.9 ± 21.3 in the Epley group and −29.5 ± 19.2 in the 360° group, corresponding to a mean difference of −0.40 (95% CI: −9.89, 9.09). Similar patterns were observed for the DHI subgroup scores (Table 4). The mean change in the VAS of vertigo score was −5.3 ± 2.8 for the Epley group and −4.4 ± 2.7 for the 360° group, corresponding to a mean difference of 0.92 (95% CI: −0.20, 2.05). The participants’ evaluation of tolerability and comfort of the treatment maneuvers were similar across the two treatment groups (Table 4).

**Table 4:** Participant-reported outcome measures.

		Epley maneuver		360° maneuver		Mean difference	(95% CI)	Cohen’s *d*
Dizziness handicap inventory								
		(*n* = 47)		(*n* = 45)				
Baseline total score	Mean ± SD	36.72	±20.66	39.42	±18.37	−2.70	(−10.81, 5.41)	−0.14
	Median [IQR]	36	[18, 54]	36	[26, 54]	–		
		(*n* = 41)		(*n* = 32)				
Post-treatment total score^a^	Mean ± SD	5.80	±9.82	8.18	±15.14	2.38	(−3.35, 8.10)	0.19
	Median [IQR]	0	[0, 8]	0	[0, 10]	–		
Change in total score	Mean ± SD	−29.85	±21.29	−29.45	±19.15	−0.40	(−9.89, 9.09)	−0.02
	Median [IQR]	−30	[−44, −14]	−28	[−28, −18]	–		
Change in subscores								
Physical change	Mean ±SD	−12.00	±6.74	−12.42	±6.50	0.42	(−2.67, 3.52)	0.06
	Median [IQR]	−12	[−18, −8]	−12	[−18, −8]	–		
Emotional change^a^	Mean ±SD	−7.27	±7.36	−5.94	±7.08	1.33	(−1.92, 4.57)	0.18
	Median [IQR]	−6	[−12, −2]	−6	[−10, −2]	–		
Functional change	Mean ± SD	−10.59	±9.14	−11.09	±8.26	0.51	(−3.58, 4.59)	0.06
	Median [IQR]	−8	[−18, −4]	−10	[−14, −4]	-		
Visual Analogue Scale of vertigo (0–10)								
		(*n* = 47)		(*n* = 43)				
Baseline score^a^	Mean ± SD	6.36	±1.92	6.02	±1.93	−0.34	(−1.12, 0.44)	−0.18
	Median [IQR]	7	[5, 8]	6	[5, 8]	–		
		(*n* = 43)		(*n* = 33)				
Post-treatment score^a^	Mean ± SD	1.02	±1.66	1.60	±2.29	0.58	(−0.25, 1.41)	0.29
	Median [IQR]	0	[0, 2]	0	[0, 3]	–		
Change in score^a^	Mean ± SD	−5.34	±2.83	−4.42	±2.67	0.92	(−0.20, 2.05)	0.33
	Median [IQR]	−5	[−7, −4]	−5	[−7, −2]	–		
Participant evaluation of treatment								
		(*n* = 46)		(*n* = 45)				
Tolerability (0–10)^a^	Mean ± SD	2.52	±2.54	2.84	±2.70	0.32	(−0.80, 1.45)	0.12
	Median [IQR]	2^a^	[0, 4]	3^a^	[0, 5]	–		
		(*n* = 46)		(*n* = 45)				
Comfort (0–10)^a^	Mean ± SD	4.72	±2.48	4.31	±2.31	−0.41	(−1.41, 0.60)	−0.17
	Median [IQR]	5^a^	[3, 6]	5^a^	[3, 5]	–		

CI, confidence interval; SD, standard deviation; IQR, interquartile range. ^a^Continuios variables with skewed distribution: mean ± SD and mean difference (95% CI) were obtained using bootstrap (with 1,000 resamples). ^b^Effect size reported as risk difference (95% CI) and Cohen’s *d* (standardized mean difference), with the Epley maneuver as the reference. Post-treatment outcomes were only assessed among participants with available follow-up data, and sample sizes therefore differ across outcomes. Please note that all mean differences had CIs that included zero, indicating no clear difference between the treatment groups. Cohen’s *d* values ranged from small to medium effect sizes between the treatment groups.

### 3.4 Adverse events

No serious adverse events related to the interventions were reported. However, minor unfavorable/unintended events, including canal shift and new contralateral BPPV, occurred in a small number of participants in both treatment groups (Table 3; Figure 3).

## 4 Discussion

### 4.1 Main findings

This randomized controlled study showed that the Epley maneuver and the 360° maneuver (both performed with an MRC) provided comparable treatment efficacy for unilateral P-BPPV, with no observed differences in any of the primary or secondary outcomes.

The majority of participants were successfully treated with a single treatment (Epley: 67%; 360°: 54%) (Table 2), and treatment success within five treatments was achieved in the majority (Epley: 90%; 360°: 78%) (Table 3). Although these numbers suggest a slightly higher efficacy of the Epley maneuver, no significant differences were detected between the two maneuvers (Tables 2, 3). A total of only three participants (Epley: *n* = 1/48, 2%; 360°: *n* = 2/46 4%) required more than five treatments (Table 3).

Notably, a non-negligible proportion of the participants classified as successfully treated still reported positional vertigo (VAS of vertigo >1) (Epley: *n* = 5/32, 16%; 360°: *n* = 4/25, 16%) despite resolution of the positional nystagmus (Table 4).

Participant-reported outcome measures (DHI and VAS of vertigo) improved substantially in both treatment groups (Table 4), with changes in the DHI score exceeding the minimally clinically important difference for DHI (≥18) (28), supporting the clinical efficacy of both treatment maneuvers.

However, the 6-month subjective recurrence rates were surprisingly high (Epley: 58%; 360°: 47%) (but comparable between the groups (risk difference: −0.11 (95% CI: −0.35, 0.13))) (Table 3), while only a minority (28%) of participants with subjective recurrence sought help from healthcare professionals.

### 4.2 Comparison with existing literature

#### 4.2.1 Treatment success rates

The treatment effect of P-BPPV has generally been well studied, primarily studies of manually performed repositioning maneuvers. However, these results are not directly transferable to MRC-based treatment due to the fundamental differences with performing of the maneuvers. Therefore, the following comparison will be restricted to studies evaluating treatment maneuvers for P-BPPV performed with the use of an MRC.

The reported success rates after a single MRC-treatment for P-BPPV vary substantially across studies. Hougaard et al. reported an overall success rate of 47% following one treatment (when applying strict criteria requiring both subjective and objective resolution), with the Epley maneuver demonstrating the highest efficacy (62%), followed by the Semont (49%) and the 360° maneuver (29%) (18). In comparison, Schuricht and Hougaard reported a similar success rate (63%) for the Epley maneuver (16), whereas Li and Epley reported a notably higher success rate (71%) for the 360° maneuver (using similar criteria of treatment success requiring both objective and subjective resolution) (32).

The success rates following a single treatment, as reported in the present study (Epley: 67%; 360°: 54%), are consistent with those of previous MRC studies. However, other MRC-based studies have even reported higher success rates following one treatment, such as Tan et al. reporting 85% following one Epley maneuver (33) and Nakayama and Epley reporting 87% (35).

Despite variability of the early treatment success rates, the overall treatment success following repeated maneuvers appears more consistent across MRC studies. Hougaard et al. reported complete resolution rates (subjective and objective) of 74% for the Epley maneuver, 62% for the Semont maneuver, and 53% for the 360° maneuver (after four treatments), with no statistically significant difference between the treatment groups (18). Considerably higher overall success rates have been reported for the 360° maneuver in other studies, ranging from 97 to 98% after three treatments (32, 35). Other studies have reported a mean number of treatments ranging from 1.38 to 2.69 for the Epley maneuver (18, 20, 33, 36) and from 1.70 to 2.33 for the 360° maneuver (18, 36).

Overall, the findings from the present study align with existing MRC-based literature, demonstrating comparable treatment success rates between the Epley and the 360° maneuvers when performed with the aid of an MRC.

However, the reported interstudy variability in treatment success rates might be explained by methodological protocol differences in relation to performing individual treatment maneuvers, especially the 360° maneuver. Hougaard et al. applied a 360° maneuver with a continuous 360° backward rotation (18), which differs from the 360° as applied in the present study (3-step maneuver) and the study by Li and Epley (2-step maneuver) (32). There exists currently no consensus on the optimal execution of the 360° maneuver, and hence several variations are used, differing in the number of positions (steps), whether the rotation is continuous or not, the duration of the positions, and angular velocity of the rotation. A recent paper by Chaure-Cordero et Martin-Sanz (2026) also highlighted the lack of standardized MRC treatment protocols as an important limitation in the current literature, emphasizing that the methodological heterogeneity remains a challenge when comparing outcomes across studies (37). Additionally, interstudy differences include differences in study populations, diagnostic criteria (subjective and/or objective findings as a prerequisite), follow-up protocols, and, importantly, the definition of treatment success.

Furthermore, it should also be acknowledged that the resolution of BPPV cannot be exclusively linked to the applied treatment maneuvers, as BPPV is known to undergo spontaneous remission in a substantial number of patients (38). Given the two-week interval between follow-up visits in the present study, spontaneous remission might have contributed to the reported treatment success rates.

#### 4.2.2 Definition of treatment success

This present study defined successful treatment according to the Bárány Society diagnostic criteria for P-BPPV (4). As these criteria require both a history of positional vertigo and BPPV characteristic positional nystagmus, participants who no longer fulfilled either diagnostic component were considered successfully treated. However, to acknowledge that subjective and objective resolution may not always occur simultaneously, subanalyses of the primary outcome were conducted to distinguish between those with complete resolution, subjective resolution, and objective resolution (Table 4). The divergence between subjective and objective resolution has important clinical implications, as assessment of positional nystagmus alone may lead to misclassification of patients with positional vertigo without positional nystagmus during the diagnostic testing (possible BPPV) (4). In addition, the applied definition of a significant positional nystagmus (≥5 consecutive beats and an aSPV of ≥3°/second) may also have influenced the categorization of objective resolution. While these were applied to ensure consistency, some participants with very low intensity positional nystagmus smaller than the predefined threshold may have been classified as successfully treated.

The mismatch between the subjective and objective outcomes is well recognized. Li and Epley reported that a higher number of participants experienced subjective resolution without corresponding objective resolution following the first treatment (32), highlighting that these two outcomes are not necessarily reported in conformity. Conversely, the present study found a different pattern, with a higher proportion of participants with objectifiable resolution of positional nystagmus only compared to those with subjective resolution of positional vertigo only (Table 4). This pattern of persistent symptoms despite objective resolution might be explained by residual dizziness, which is frequently reported condition among patients following successful BPPV treatment (39, 40). Residual dizziness is considered as multifactorial and may involve delayed vestibular compensation, persistent sensory mismatch between vestibular, visual, and somatosensory inputs, or psychosocial factors that influence the perception of vertigo (39). Although the extensive head movements associated with the use of an MRC might contribute to transient vestibular sensory mismatch, the present study was not designed to investigate the underlying mechanisms of persistent symptoms. Therefore, it cannot be determined by the present study whether the reported residual symptoms were related to the use of an MRC itself. Although data on residual dizziness were not systematically collected in the present study, we observed that a substantial number of participants reporting this following successful treatment. Conversely, the persistence of low-intensity positional nystagmus despite symptom resolution may, in part, be explained by the presence of physiological positional nystagmus, which has been shown to occur in the majority of healthy individuals (41, 42).

Furthermore, a previous study showed that vertiginous symptoms, measured by the VAS of vertigo (as used in the present study to evaluate subjective resolution), do not necessarily reach zero after successful treatment (43), suggesting that complete subjective resolution may be too strict an endpoint.

In summary, these findings emphasize that treatment success rates are highly dependent on the definition used and that interstudy comparisons should be interpreted with caution (35).

#### 4.2.3 Recurrence rates

The natural history of BPPV treatment is characterized by relatively high recurrence rates. However, the reported rates vary widely across studies, ranging from 14 to 48% depending on the length of follow-up and study design (44). Most recurrences seem to appear within the first year following successful treatment (45). In studies focusing on MRC-based treatment, short-term recurrence rates for P-BPPV are reported to range from 18 to 27% (43, 46). Compared with these numbers, the subjective recurrence rates, as observed in the present study, were rather high (Epley: 58%; 360°: 47%) during the 6-month follow-up period. This discrepancy is most likely explained by methodological differences, particularly the use of self-reported recurrence without confirmation by clinical examination in the present study. Therefore, it remains uncertain whether the reported subjective recurrence represents true BPPV recurrences, other vestibular conditions, residual dizziness, or simply non-specific and uncharacteristic symptoms of dizziness and/or vertigo. This uncertainty is further supported by the fact that only 27% of the participants, who reported subjective recurrence, sought help from healthcare professionals. However, this rather low number of participants with a suspected recurrence, who actually sought help from a professional, should also be interpreted with caution due to the self-limiting nature of BPPV. Given the relatively high number of spontaneous remissions of BPPV (38) one could speculate that an unknown proportion of true BPPV recurrences may remain undiagnosed in studies relying solely on clinically confirmed cases. Consequently, these studies may underestimate the true recurrence rate, whereas questionnaire-based studies may overestimate it.

Despite these differences in recurrence rates, the absence of differences between the treatment groups in the present study was consistent with previous findings (43, 46). This might suggest that BPPV recurrence is not linked to the specific repositioning maneuver applied but is more likely related to variables associated with the underlying pathophysiology and patient-related factors (44).

#### 4.2.4 Participant-reported outcome measures

Participant-reported outcome measures, including DHI and VAS of vertigo, are increasingly used as meaningful and important measures of the treatment efficacy. Previous studies have reported significant improvement of total DHI-scores following successful treatment of P-BPPV (16, 18, 46). Likewise, West et al. reported a significant reduction in the score of both the DHI and VAS of vertigo following successful MRC treatment (43). However, in this study, these measures did not reach zero for the majority of patients, reflecting residual symptoms (43). The findings in the present study align with these previous results, demonstrating clinically meaningful improvements (change in DHI total score ≥18) (28) after successful treatment (Table 4; Figures 5, 6).

Additionally, both treatment maneuvers were well tolerated in the present study, which also aligns with previous reported results with high tolerability and patient satisfaction with the 360° maneuver with an MRC (32). Notably, this study (by Li and Epley (32)) also reported that patient-reported comfort improved with repetitive treatments, suggesting that being familiar with the MRC procedure may positively influence the patient experience.

### 4.3 Strengths and limitations

The main strength of this study was its prospective, randomized design, which reduces selection bias and allows us to directly compare two separate treatment maneuvers. The two treatment maneuvers were performed by three clinicians, introducing a risk of inter-clinician variability. This risk was mitigated through standardized training based on a detailed trial protocol, and, whenever possible, participants were assessed and treated by the same clinician across visits to enhance consistency.

In addition, all diagnostics and treatments in this study were conducted with the aid of an MRC in combination with VNG goggles. This ensured standardization and reproducibility of the head orientation during the positional maneuvers (due to the preset positions of the MRC) as well as the improved quantification and recording of positional nystagmus provided by the VNG goggles. Another strength of the study is the systematic, repeated follow-up evaluation of subjective recurrence(s) by fulfillment of questionnaires at two predetermined time points (3 and 6 months following successful treatment). This approach reduced the risk of recall bias compared to longer recall periods.

Several limitations should also be considered when interpreting the results of this trial. In relation to the study design, blinding of participants or clinicians was not feasible given the nature of the interventions. Although the study sample was relatively large (*n* = 102), the study remained underpowered relative to the target sample size (*n* = 128) as defined by the *a priori* power calculation. This increases the risk of a type II error (failing to detect a true difference) and limits the study’s ability to detect smaller differences between the treatment groups. However, given the relatively large study sample, one could speculate whether such differences would have been clinically relevant.

Another important methodological consideration related to our classification of unsuccessful treatment, including participants with the need for more than five treatments and those experiencing canal shift of new contralateral BPPV. This approach both excluded information on the actual number of treatments required for those participants (*n* = 3) and may have led to an overestimation of unsuccessful treatment rates as these scenarios may reflect complex BPPVs rather than true treatment failure.

Since data on subjective recurrence and participant-reported outcome measures were collected via electronic questionnaires, these data could be affected by a non-response bias among older patients. Additionally, non-response might be influenced by other factors such as age, symptom severity, recurrence status, and overall motivation to participate. Participants experiencing recurrence of vertiginous symptoms may be more or less likely to respond, potentially biasing the results in either direction.

Although subjective recurrence was assessed after 3 and 6 months, a long-term follow-up was not conducted. Consequently, this study cannot provide insights into long-term recurrence patterns in the two treatment subgroups. Additionally, the reported subjective recurrence rates were not systematically verified by an in-person evaluation of BPPV-characteristic positional nystagmus. Moreover, given the small number of cases with cupulolithiasis subtype BPPV (Epley: *n* = 4/48; 360°: *n* = 4/46), a subgroup analysis of BPPV subtype was not performed. This may, in theory, have affected the results, as cupulolithiasis is believed to be more resistant to treatment (20).

The demographic characteristics of the study population align with previously reported data in the literature (1–3), supporting the generalizability of the findings. However, it is important to note that most participants in this study were referred by their general practitioner, hereby likely representing a predominantly unselected population with uncomplicated BPPV. In contrast, MRCs are predominantly available at tertiary dizziness clinics, where referral pathways often involve more specialized units, such as private ENT clinics or other hospital departments (may include more complex or treatment refractory BPPVs). Consequently, the findings of the present study may be most directly applicable to patients with uncomplicated P-BPPV, and generalization to more specialized populations should be made with caution.

### 4.4 Implications of results and future research

The findings of the present study can be applied to clinicians who use an MRC to treat adults diagnosed with P-BPPV. The results suggest that both the Epley- and the 360° maneuvers have comparable efficacy, with no clinically meaningful differences in the outcomes. However, this finding should be interpreted in the context of the MRC’s standardized execution of the Epley maneuver and may (most likely) not be directly transferable to the manually performed Epley maneuver, which is associated with substantial variability when performed without any guidance system (47). Thus, the observed equivalence between the Epley- and 360° maneuvers most likely reflects standardized execution by the MRC rather than true maneuver independence. Future research should focus on the clinical application of MRCs rather than comparing individual treatment maneuvers. Specifically, studies are recommended to investigate patient-specific characteristics (e.g., first-episode vs. recurrent or refractory BPPV) to optimize the treatment protocols. In addition, long-term follow-up studies with objective verification of BPPV recurrences would provide a better understanding of BPPV relapse patterns and BPPV-related residual dizziness.

## 5 Conclusion

This randomized controlled study examined treatment of unilateral posterior BPPV with the use of a mechanical rotation chair and dermonstrated a comparable treatment efficacy between the Epley maneuver and the 360° maneuver across both objective and subjective (patient-reported outcome measures) outcomes. However, the study did not reach the planned sample size and was therefore underpowered relative to the original power calculation. These findings should therefore be interpreted with caution and future adequately powered randomized controlled studies are needed to confirm these findings, optimize treatment protocols by identifying patient subgroups that may benefit from specific maneuvers, and evaluate long-term outcomes and recurrence patterns.

## Data Availability

The raw data supporting the conclusions of this article will be made available by the authors, without undue reservation.
