# Supplementary material for: Epley maneuver versus 360° maneuver for treatment of posterior canal benign paroxysmal positional vertigo in a mechanical rotation chair: a randomized controlled trial
Source: Front Neurol. 2026 Sep 8;17:1909757. doi: 10.3389/fneur.2026.1909757 (PMC13598661; doi:10.3389/fneur.2026.1909757)
Supplement: Supplementary file 1 [file Table_1.DOCX]

Supplementary Material

| **Table A. Overview of study period, including enrollment and data collection points.** | | | | | | | | |
| --- | --- | --- | --- | --- | --- | --- | --- | --- |
|  | Visit | | | | | | Months after successful treatment | |
| TIMEPOINT | 1. | 2. | … | 5 | Last visit | 3 | | 6 |
| ENROLMENT: |  |  |  |  |  |  | |  |
| Eligibility screen | X |  |  |  |  |  | |  |
| Informed consent | X |  |  |  |  |  | |  |
| Allocation | X |  |  |  |  |  | |  |
| INTERVENTIONS: |  |  |  |  |  |  | |  |
| Epley’s maneuver | X | X | X | X |  |  | |  |
| 360° maneuver | X | X | X | X |  |  | |  |
| ASSESSMENTS: |  |  |  |  |  |  | |  |
| Baseline data | x |  |  |  |  |  | |  |
| VAS of vertigo | X | X | X | X | X |  | |  |
| BPPV diagnostics with MRC |  | X | X | X | X |  | |  |
| BPPV treatment (protocol) | X | X | X | X |  |  | |  |
| DHI | X |  |  |  | X* |  | |  |
| Follow-up questionnaire (BPPV recurrence) |  |  |  |  |  | X* | | X* |
| VAS: Visual Analogue Scale; MRC: mechanical rotation chair; DHI: Dizziness Handicap Inventory.  *Only including participants with successful treatment. | | | | | | | | |
